# Supplementary material for: Systematic Dimensionality Reduction for Quantum Walks: Optimal Spatial Search and Transport on Non-Regular Graphs
Source: Sci Rep. 2015 Sep 2;5:13304. doi: 10.1038/srep13304 (PMC4557070; doi:10.1038/srep13304)
Supplement: Supplementary Information [file srep13304-s1.pdf]

Supplementary Information
Systematic Dimensionality Reduction for Quantum Walks: Optimal Spatial Search and Transport on Non-Regular Graphs

Leonardo Novo[1,2,a],[*] Shantanav Chakraborty[1,2,a], Masoud Mohseni[3], Hartmut Neven[3], and Yasser Omar[1,2,4]
[1]*Physics of Information Group, Instituto de Telecommunicações, Portugal*
[2]*Instituto Superior Técnico, Universidade de Lisboa, Portugal*
[3]*Google Inc., Venice, CA 90291, USA and*
[4]*CEMAPRE, ISEG, Universidade de Lisboa, Portugal*
([a] Both authors have equal contribution)
(Dated: April 22, 2015)

PROOF OF THE EQUALITY OF $\mathcal{I}(H, |w\rangle)$ AND $\Lambda(H, |w\rangle)$

In [1], the authors calculate the transport efficiency of structures in the no disorder, no dephasing regime, by calculating the overlap of the initial state with the subspace spanned by the eigenstates of the Hamiltonian that have a non-zero overlap with the trap node $|w\rangle$. Let this subspace be denoted by $\Lambda(H, |w\rangle)$. Here we prove that this subspace is equal to the space containing the trap node $|w\rangle$ and that is invariant under the unitary evolution, denoted by $\mathcal{I}(H, |w\rangle)$.

Now, $\Lambda(H, |w\rangle) = \text{span}(\{|\lambda_1\rangle, \ldots, |\lambda_m\rangle\})$ where, $|\lambda_k\rangle$ are the minimum number of eigenstates of H with $H|\lambda_k\rangle = \lambda_k|\lambda_k\rangle$, such that $\langle\lambda_k|w\rangle \neq 0$ (there will be $N-m$ eigenstates $|\lambda_k\rangle$ with $\langle\lambda_k|w\rangle = 0$ for $k \in \{m+1, \ldots, N\}$). Here by *minimum* number of eigenstates, it is meant that in the case of degenerate eigenspaces, more than one eigenstate can have a non-zero overlap with $|w\rangle$. In such a scenario, this ambiguity is resolved by choosing the eigenvector from this degenerate eigenspace that has the maximum possible overlap with $|w\rangle$ and orthogonalize all the other vectors within this eigenspace with respect to it. This implies that the remaining eigenvectors is the degenerate space would have zero overlap with $|w\rangle$ post orthogonalization. This procedure is explained in [1] where this subspace is referred to as the non-invariant subspace and its calculation provides a simple way of obtaining the efficiency of transport to a trapping site on the graph (in the absence of dephasing and losses).

Let us first assume that $|\mathcal{I}(H, |w\rangle)| = m_1$ and $|\Lambda(H, |w\rangle)| = m_2$. It is simple to see that $\mathcal{I}(H, |w\rangle) \in \Lambda(H, |w\rangle)$ by expressing the state $H^i|w\rangle$ as

$$H^i|w\rangle = \sum_{k=1}^{N} \langle\lambda_k|w\rangle H^i|\lambda_k\rangle \tag{1}$$

$$= \sum_{k=1}^{m_2} \langle\lambda_k|w\rangle H^i|\lambda_k\rangle \tag{2}$$

$$= \sum_{k=1}^{m_2} \langle\lambda_k|w\rangle {\lambda_k}^i|\lambda_k\rangle, \tag{3}$$

where in the first step we used that $\langle\lambda_k|w\rangle = 0$ for $k \in \{m_2+1, \ldots, N\}$. Since the states $H^i|w\rangle$ span $\mathcal{I}(H, |w\rangle)$ and each of these states can be expressed in terms of elements of $\Lambda(H, |w\rangle)$, we conclude that $\mathcal{I}(H, |w\rangle) \in \Lambda(H, |w\rangle)$ and $m_2 \geq m_1$.

Now, it remains to show that each element of $\Lambda(H, |w\rangle)$ can be expressed as

$$|\lambda_j\rangle = \sum_{i=1}^{m_1} c_{ji} H^{i-1}|w\rangle \tag{4}$$

$$= \sum_{i=1}^{m_1} \sum_{k=1}^{m2} c_{ji} \langle\lambda_k|w\rangle {\lambda_k}^{i-1}|\lambda_k\rangle \tag{5}$$

[*] Correspondence to: lnovo@lx.it.pt

where c_{ji} are coefficients and $j \in \{1, \ldots, m_2\}$. For this to happen we obtain the condition

$$\sum_{i=1}^{m_1} c_{ji} \langle \lambda_k | w \rangle {\lambda_k}^{i-1} = \delta_{jk}. \tag{6}$$

Defining the matrix $M_{ik} = \langle \lambda_k | w \rangle {\lambda_k}^{i-1}$, Eq. (6) is equivalent to the condition $\sum_{i=1}^{m_1} c_{ji} M_{ik} = \delta_{jk}$. Thus, M must be an invertible $m \times m$ matrix, so we must have $m_1 = m_2 = m$. To show that M is always invertible we show that $det(M) \neq 0$. For this we define two $m \times m$ matrices, $V_{ij} = {\lambda_j}^{i-1}$ and the diagonal matrix $D_{ij} = \delta_{ij} \langle \lambda_j | w \rangle$ such that $M = DV$. Because $\langle \lambda_j | w \rangle \neq 0$ for $k \in \{1, \ldots, m\}$ we conclude that $\det(D) \neq 0$. Also, V is of the Vandermonde form so its determinant is given by $\det(V) = \prod_{1 \leq i < j \leq m} (\lambda_i - \lambda_j)$. Because all $|\lambda_k\rangle$ belong to different eigenspaces for $k \in \{1, \ldots, m\}$, all λ_k are different from each other and the Vandermonde determinant is not zero. Thus $\det(M) = \det(D)\det(V) \neq 0$ so M is invertible. This completes the proof that $\mathcal{I}(H, |w\rangle) = \Lambda(H, |w\rangle)$.

Thus, the subspace comprising of the eigenstates of the Hamiltonian having a non-zero overlap with $|w\rangle$ is the same as the one containing $|w\rangle$ and is invariant under the unitary evolution. The advantage of working with the latter subspace is that, one does not need to diagonalize the Hamiltonian to obtain this space.

OPTIMAL SEARCH ON A COMPLETE GRAPH WITH k BROKEN LINKS SUCH THAT A LINK CONNECTED TO THE SOLUTION NODE IS BROKEN

From a complete graph, k links are broken in a manner such that at most one link is broken per node, including the solution state, and thus, $k \leq \frac{N}{2}$. The system Hamiltonian evolves in a four dimensional subspace as we shall show subsequently. We assume that the link connected to the marked state that is broken is represented by (w, a). The remaining $k-1$ broken links are not connected to w and so the set of all these broken links are represented by E_{broken}, whose cardinality is $2k-2$. Also, let V_{broken} be the set of nodes comprising of these $k-1$ broken links. Now, let

$$|s_{k-1}\rangle = \frac{1}{\sqrt{2k-2}} \sum_{l \in V_{\text{broken}}} |l\rangle, \tag{7}$$

be the equal superposition of the $2k-2$ nodes corresponding to the $k-1$ broken links that that are not connected to $|w\rangle$. Also let,

$$|s_{\overline{k-1}}\rangle = \frac{1}{\sqrt{N-2k}} \sum_{\substack{g \notin V_{\text{broken}}, \\ g \notin \{|w\rangle, |a\rangle\}}} |g\rangle, \tag{8}$$

be the equal superposition of the nodes that have degree N, i.e., they do not correspond to any broken link.

Projecting on to the space $\mathcal{I}(H, |w\rangle)$ gives the reduced Hamiltonian in the basis $\{|w\rangle, |r_{\bar{a}}\rangle, |r_{\bar{a}}^{\perp}\rangle, |a\rangle\}$, where

$$|r_{\bar{a}}\rangle = \sqrt{\frac{N-2k}{N-2}} |s_{\overline{k-1}}\rangle + \sqrt{\frac{2k-2}{N-2}} |s_{k-1}\rangle, \tag{9}$$

and,

$$|r_{\bar{a}}^{\perp}\rangle = \sqrt{\frac{2k-2}{N-2}} |s_{\overline{k-1}}\rangle - \sqrt{\frac{N-2k}{N-2}} |s_{k-1}\rangle. \tag{10}$$

The search Hamiltonian is thus,

$$H_{\text{search}} = -\gamma \begin{bmatrix} \frac{1}{\gamma} & \sqrt{N-2} & 0 & 0 \\ \sqrt{N-2} & N+5-\frac{2k+2}{N-2} & \frac{\sqrt{(k-1)(N-2k)}}{N-2} & \sqrt{N-2} \\ 0 & \frac{\sqrt{(k-1)(N-2k)}}{N-2} & -10+\frac{2k+2}{N-2} & 0 \\ 0 & \sqrt{N-2} & 0 & 0 \end{bmatrix}. \tag{11}$$

The initial superposition of states can be approximated to be $|r_{\bar{a}}\rangle$ in the limit of large N. Now, let $k = \alpha N$, where, $0 \leq \alpha \leq \frac{1}{2}$ and N being large. Thus H_{search} becomes

$$H_{\text{search}} = -\gamma \begin{bmatrix} \frac{1}{\gamma} & \sqrt{N} & 0 & 0 \\ \sqrt{N} & N & \sqrt{\alpha(1-2\alpha)} & \sqrt{N} \\ 0 & \sqrt{\alpha(1-2\alpha)} & 2\alpha & 0 \\ 0 & \sqrt{N} & 0 & 0 \end{bmatrix}. \tag{12}$$

Degenerate perturbation theory enables us to separate H_{search} into $H^{(0)}$, $H^{(1)}$ and $H^{(2)}$ of terms of order $\mathcal{O}(1)$, $\mathcal{O}(\frac{1}{\sqrt{N}})$ and $\mathcal{O}(\frac{1}{N})$ respectively. We find the critical value of $\gamma = \frac{1}{N}$ and the eigenvalues of $H^{(0)} + H^{(1)}$ to be $E_{\pm} = 1 \pm \frac{1}{\sqrt{N}}$. Thus the running time is again $T = \frac{\pi\sqrt{N}}{2}$, which is the optimal value.

TRANSPORT EFFICIENCY FOR THE COMPONENT OF THE INITIAL CONDITION WITHIN $\mathcal{I}(H, |trap\rangle)$

Here we show that if the initial state $|\psi(0)\rangle \in \mathcal{I}(H, |trap\rangle)$, the transport efficiency is one. The efficiency of transporting an exciton from a starting node to the trap is given by

$$\eta = 2\kappa \int_0^\infty dt \ \langle\psi(t)|trap\rangle \langle trap|\psi(t)\rangle \,. \tag{13}$$

Now,

$$\frac{d}{dt}(\langle\psi(t)|\psi(t)\rangle) = \langle\dot{\psi(t)}|\psi(t)\rangle + \langle\psi(t)|\dot{\psi(t)}\rangle \,. \tag{14}$$

Using the Schrödinger equation to replace $|\dot{\psi(t)}\rangle$,

$$\frac{d}{dt}(\langle\psi(t)|\psi(t)\rangle) = -2\kappa \langle\psi(t)|trap\rangle \langle trap|\psi(t)\rangle \,. \tag{15}$$

Thus,

$$\begin{aligned} \eta &= \int_0^\infty d(\langle\psi(t)|\psi(t)\rangle) \\ &= \langle\psi(0)|\psi(0)\rangle - \langle\psi(\infty)|\psi(\infty)\rangle \\ &= 1. \end{aligned} \tag{16}$$

This is using the fact that the anti-hermitian term of the Hamiltonian reduces the norm of the state $|\psi(t)\rangle$ at the rate κ and for $t \to \infty$, the component of the wave function within $\mathcal{I}(H, |trap\rangle)$ gets absorbed completely and hence $\langle\psi(\infty)|\psi(\infty)\rangle = 0$.

This implies that to calculate the transport efficiency of an exciton starting from an initial state to a trap, it suffices to calculate the overlap of the initial state with $\mathcal{I}(H, |w\rangle)$. The component of the exciton outside this subspace will not get absorbed by the trap, but rather will remain in the network.

---

[1] Filippo Caruso, Alex W Chin, Animesh Datta, Susana F Huelga, and Martin B Plenio. Highly efficient energy excitation transfer in light-harvesting complexes: The fundamental role of noise-assisted transport. *The Journal of Chemical Physics*, **131**:105106, 2009.
